# Elevated oxysterol levels in human and mouse livers reflect nonalcoholic steatohepatitis[S]

**DOI:** 10.1194/jlr.M093229

**Published:** 2019-05-21

**Authors:** Tina Raselli, Tom Hearn, Annika Wyss, Kirstin Atrott, Alain Peter, Isabelle Frey-Wagner, Marianne R. Spalinger, Ewerton M. Maggio, Andreas W. Sailer, Johannes Schmitt, Philipp Schreiner, Anja Moncsek, Joachim Mertens, Michael Scharl, William J. Griffiths, Marco Bueter, Andreas Geier, Gerhard Rogler, Yuqin Wang, Benjamin Misselwitz

**Affiliations:** Department of Gastroenterology and Hepatology,* University Hospital Zurich and University of Zurich, Zurich, Switzerland; Department of Visceral Surgery§§ University Hospital Zurich and University of Zurich, Zurich, Switzerland; Institute for Surgical Pathology§ University Hospital Zurich and University of Zurich, Zurich, Switzerland; Swansea University Medical School † Singleton Park, Swansea, United Kingdom; Chemical Biology and Therapeutics,** Novartis Institutes for BioMedical Research, Basel, Switzerland; Division of Hepatology†† Department of Internal Medicine II, University Hospital Würzburg, Würzburg, Germany; Department of Visceral Surgery and Medicine,*** Inselspital Bern and Bern University, Bern, Switzerland

**Keywords:** nonalcoholic fatty liver disease, Epstein-Barr virus-induced gene 2, cholesterol 25 hydroxylase, 25-hydroxycholesterol 7α-hydroxylase, mouse feeding model

## Abstract

Nonalcoholic steatohepatitis (NASH), a primary cause of liver disease, leads to complications such as fibrosis, cirrhosis, and carcinoma, but the pathophysiology of NASH is incompletely understood. Epstein-Barr virus-induced G protein-coupled receptor 2 (EBI2) and its oxysterol ligand 7α,25-dihydroxycholesterol (7α,25-diHC) are recently discovered immune regulators. Several lines of evidence suggest a role of oxysterols in NASH pathogenesis, but rigorous testing has not been performed. We measured oxysterol levels in the livers of NASH patients by LC-MS and tested the role of the EBI2-7α,25-diHC system in a murine feeding model of NASH. Free oxysterol profiling in livers from NASH patients revealed a pronounced increase in 24- and 7-hydroxylated oxysterols in NASH compared with controls. Levels of 24- and 7-hydroxylated oxysterols correlated with histological NASH activity. Histological analysis of murine liver samples demonstrated ballooning and liver inflammation. No significant genotype-related differences were observed in *Ebi2^−/−^* mice and mice with defects in the 7α,25-diHC synthesizing enzymes CH25H and CYP7B1 compared with wild-type littermate controls, arguing against an essential role of these genes in NASH pathogenesis. Elevated 24- and 7-hydroxylated oxysterol levels were confirmed in murine NASH liver samples. Our results suggest increased bile acid synthesis in NASH samples, as judged by the enhanced level of 7α-hydroxycholest-4-en-3-one and impaired 24*S*-hydroxycholesterol metabolism as characteristic biochemical changes in livers affected by NASH.

Nonalcoholic fatty liver disease (NAFLD) is the most frequent liver disease in industrialized countries (1). The prevalence of NAFLD ranges from 11% to 46%, with a continuous increase in incidence within the last two decades (2–7). NAFLD reflects liver injury in the setting of overnutrition, sedentary life style, obesity, and metabolic syndrome. NAFLD comprises a clinical spectrum ranging from benign liver steatosis to nonalcoholic steatohepatitis (NASH), with its associated complications liver fibrosis, liver cirrhosis, and hepatocellular carcinoma. Because therapeutic options for viral hepatitis have improved dramatically, NASH is expected to be the main cause of chronic liver disease in the years to come (5). Despite a large number of ongoing clinical trials, no medical treatment for NASH has been established to date (8, 9).

The pathogenesis of NASH is still incompletely understood; current concepts implicate lipids and toxic lipid metabolites, reactive oxygen species, or products of the intestinal microbiota as causes of liver injury, which would lead to liver infiltration by immune cells and continued destruction of liver tissue (10–12). However, the pathway from tissue metabolic derangement to liver inflammation and fibrosis is complex and not yet sufficiently clarified.

Oxysterols are oxidized cholesterol molecules that are formed in the early steps of cholesterol metabolism and bile acid synthesis. They are regulators of lipid metabolism with important metabolic functions, including the induction of key enzymes of the bile acid pathway, stimulation of reverse cholesterol transport, and regulation of hepatic cholesterol and fatty acid synthesis (13). Importantly, metabolic changes in NAFLD patients are accompanied by increased serum concentrations of oxysterols such as 25-hydroxycholesterol (25-HC) and 27-hydroxycholesterol [27-HC; synonym: (25*R*)26-hydroxycholesterol] (14). However, the full extent of changes in oxysterol metabolism in liver tissue over the spectrum of disease from bland steatosis to NASH and fibrosis has been insufficiently studied.

It is currently unclear whether oxysterols are active players or bystanders in NASH pathogenesis, but some arguments for an active role have been put forward: In a murine model of NASH, the inhibition of 27-HC synthesis (due to knockdown of the enzyme CYP27A1) resulted in stronger NASH activity compared with wild-type and subcutaneous application of 27-HC, which substantially decreased liver inflammation (15). The protective effects of oxysterols are not limited to 27-HC; in animal models of obesity or diabetes mellitus, the enzyme CYP7B1 (mediating 7α-hydroxylation of oxysterols) is dramatically downregulated (16–18). In line with these observations, the transient hepatic overexpression of CYP7B1 in obese mice improved hepatic steatosis and metabolic syndrome (16).

Oxysterols have also been implicated as critical immune regulatory molecules (19–21). 25-HC is part of the rapid innate immune response and can induce macrophage activation (19, 22, 23), T-cell differentiation (20), and the production of IL-8 (24–26) and IL-6 (23). It has also been shown to have strong antiviral activity against many enveloped viruses (21, 27, 28). Furthermore, 25-HC has been shown to inhibit the activation of the DNA sensor protein AIM2, preventing spurious AIM2 inflammasome activation (29).

The oxysterol 7α,25-dihydroxycholesterol (7α,25-diHC) was discovered as a ligand for the Epstein-Barr virus-induced G protein-coupled receptor 2 (EBI2; also known as GPR183) (30, 31). 7α,25-diHC is produced by 25-hydroxylation of cholesterol by CH25H followed by 7α-hydroxylation by CYP7B1 (32). The EBI2-7α,25-diHC system can stimulate the migration of B-, T-, and dendritic cells and is crucial for an early antibody response against T-cell-dependent antigens (33). EBI2-dependent immune cell migration is a fundamental immunological mechanism in diverse processes, including IgG production (30), asthma (34) and encephalitis (35, 36).

EBI2 is also involved in intestinal inflammation: EBI2 was described as an inflammatory bowel disease risk gene (37), and recent data demonstrate a role of EBI2 in the induction of colitis (38, 39). EBI2 and its oxysterol ligands thereby promote the development of lymphoid structures in the large intestine in health and accumulation of lymphoid tissue in inflammation, potentially facilitating an influx of immune cells into the gut.

Taken together, oxysterols have regulatory effects for fat and cholesterol metabolism as well as the immune system, rendering these molecules interesting candidates as key mediators of NASH pathogenesis. We therefore set out to analyze free oxysterol levels in human NASH and performed a systematic study using a physiological murine feeding model and knockout mice of key players of the EBI2 and 7α,25-diHC axis to rigorously assess a role of EBI2 and its ligand in the pathogenesis of NASH.

## MATERIALS AND METHODS

### Patients

Bariatric patients were included in the study when the following inclusion criteria were met: age ≥18 years; BMI >35 kg/m^2^; and failed attempt for weight reduction ≥2 years or ≥1 year if BMI >50 kg/m^2^. Exclusion criteria were pregnancy, inflammatory bowel disease, liver cirrhosis Child B/C, or the presence of another serious medical, surgical, or psychiatric condition precluding bariatric surgery. All patients provided written informed consent prior to being included in this study. The study was performed according to the principles of the Helsinki Declaration and approved by the ethics committee of Zurich County (KEK-ZH-Nr. 2012-0260).

Baseline characteristics were collected the day of bariatric surgery (Roux-en-Y gastric bypass or gastric sleeve surgery). A liver biopsy was acquired intraoperatively using a 18 G Tru-Cut biopsy needle that yielded 4–16 mg liver tissue, which was immediately frozen in liquid nitrogen. Four biopsy specimens were obtained from the resected specimen of liver resections with informed consent and ethics approval (KEK-ZH-no. 2013-0503).

### Animals

The animal experimental protocol was approved by the local animal welfare authority (Tierschutzkommision Zürich, Zurich, Switzerland; registration number ZH 50/2013).

CH25H-deficient mice (*Ch25h^−/−^*) in a C57BL/6 background and EBI2-deficient mice (*Ebi2^−/−^*) in a C57BL/6 background were provided by Novartis Institutes for BioMedical Research (40, 41). CYP7B1-deficient mice (*Cyp7b1^−/−^*) were purchased from Jackson Laboratories. All mice were bred in our animal facility with pure C57BL/6 mice to generate heterozygous mice (*Ch25h^+/−^*, *Ebi2^+/−^*, or *Cyp7b1^+/−^*). Heterozygous mice were subsequently crossed with each other to obtain wild-type (*Ch25h^+/+^*, *Ebi2*^+/+^, or *Cyp7b1*^+/+^) and knockout (*Ch25h^−/−^*, *Ebi2^−/−^*, or *Cyp7b1^−/−^*) littermates. The mice were housed under specific pathogen-free conditions in individually ventilated cages. Eight-week-old male littermates were used for all studies. NAFLD was induced by ad libitum feeding of a high-fat, high-cholesterol diet (ssniff Spezialdiäten GmbH) and high-fructose corn-syrup equivalent (55% fructose and 45% glucose) (42) in drinking water at a concentration of 42 g/l for either 10 or 20 weeks. Control mice received standard laboratory chow (Provimi Kliba) and water ad libitum.

### Sterol extraction, derivatization, and LC-MS

The method for sterol extraction, derivatization, and LC-MS has been previously described in detail (43). The sterols measured here are free nonesterified cholesterol and oxysterols, which are different from total sterols, that is, the sum of esterified and nonesterified molecules referred to below. We opted for measuring free oxysterols because free oxysterols better reflect the biological activity. Briefly, liver tissue was homogenized in ethanol containing deuterated internal standards; oxysterols were separated from cholesterol by C18 solid-phase extraction and derivatized with Girard P reagent (US Biological) with or without prior oxidation of 3β-hydroxy to 3-oxo groups. The oxysterols were analyzed using LC-MS^n^ (multistage fragmentation) on an Orbitrap Elite mass spectrometer.

The following oxysterols were quantified: 7α-hydroxycholesterol (7α-HC, cholest-5-ene-3β,7α-diol); 7α-hydroxycholest-4-en-3-one (7α-HCO); 22R-hydroxycholesterol (22R-HC, cholest-5-en-3β,22R-diol), 27-HC (cholest-5-ene-3β,(25R)26-diol); 25-HC (cholest-5-ene-3β,25-diol); 24S-hydroxycholesterol (24S-HC, cholest-5-ene-3β,24*S*-diol); 24R-hydroxycholesterol (24R-HC, cholest-5-ene-3β,24*R*-diol); 7β-hydroxycholesterol (7β-HC, cholest-5-ene-3β,7β-diol); 6-hydroxycholesterol (6-HC, cholest-5-ene-3β,6-diol); 7α,12α-dihydroxycholesterol (7α,12α-diHC, cholest-5-ene-3β,7α,12α-triol); 7α,12α-dihydoxycholest-4-en-3-one (7α,12α-diHCO); 7α,27-dihydroxycholesterol (7α,27-diHC, cholest-5-ene-3β,7α,(25*R*)26-triol); 7α,27-dihydroxycholest-4-en-3-one (7α,27-diHCO; 7α,(25*R*)26-dihydroxycholest-4-en-3-one); 7α,25-diHC (cholest-5-ene-3β,7α,25-triol); 20R,22R-dihydroxycholesterol (20R,22R-diHC, cholest-5-ene-3β,20R,22R-triol); and 7α,24S-dihydroxycholesterol (7α,24S-diHC, cholest-5-ene-3β,7α,24*S*-triol) in addition to cholesterol and 3β-hydroxycholest-5-en-(25*R*)26-oic acid.

As standards for measurements, 7α,12α-dihydroxycholest-4-en-3-one was purchased from Toronto Research Chemicals. 20*R*,22*R*-diHC was a gift from Ingemar Björkhem (Karolinska Institute). All other oxysterol standards were purchased from Avanti Polar Lipids.

### Measurement of biochemical parameters in patient serum

Aspartate transaminase (AST), alanine transaminase (ALT), and total cholesterol (sum of cholesterol esters and free cholesterol) were measured using Fuji Dri-Chem slides (Fujifilm) on a Dri-Chem NX500 device (Fujifilm).

### RNA isolation, cDNA synthesis, and real-time PCR

Total RNA was isolated using the RNeasy Plus Mini Kit (QIAGEN). Lysis buffer from the kit was added to snap-frozen resections, and samples were shredded in M tubes (Miltenyi Biotec) in a gentleMACS tissue homogenizer (Miltenyi Biotec). Total RNA was prepared according to the manufacturer’s instructions. On-column DNase digestion with RDD buffer (QIAGEN) was performed for 15 min at room temperature. RNA concentration was determined by absorbance at 260 and 280 nm. cDNA (cDNA) synthesis was performed using a high-capacity cDNA reverse-transcription kit (Applied Biosystems) following the manufacturer’s instructions. Real-time PCR was performed using the TaqMan Fast Universal Master Mix (Applied Biosystems) on a Fast 7900HT real-time PCR system and the SDS software (Applied Biosystems). The real-time PCR started with an initial enzyme activation step (5 min; 95°C), followed by 45 cycles consisting of a denaturing (95°C; 15 s) and annealing/extending (60°C; 1 min) step. For each sample, triplicates were measured, and hypoxanthine-guanine phosphoribosyltransferase was used as endogenous control. Results were analyzed by the ΔΔCT method. All gene expression assays were obtained from Life Technologies.

### Histology

Livers were fixed in 4% paraformaldehyde and embedded in paraffin. Histological scoring for steatosis, cellular hypertrophy, and necroinflammation was performed on hematoxylin/eosin (H/E)-stained sections of the median liver lobe as described previously (44). H/E and Sirius Red staining was performed on 3 µm paraffin sections, whereas for Oil Red O staining 5 µm cryosections were used. The sections were examined using an AxioCam HRc (Zeiss) on a Zeiss Axio Imager.Z2 microscope with AxioVision version 4.8.2 software.

### Flow cytometry analysis

After the removal of the median and the left liver lobes for RNA isolation and histological and oxysterol extraction, the liver was perfused with 0.1% Collagenase IV (Sigma-Aldrich). The liver was then removed and minced, and intrahepatic mononuclear cells were purified using a Ficoll gradient. The LIVE/DEAD Fixable Dead Stain Kit (Life Technologies) was used for the exclusion of dead cells. Data acquisition was performed on a FACSCanto II (BD Biosciences), and FlowJo (FlowJo LLC) software was used for data analysis.

### Statistics

Unless otherwise indicated, data are presented as means ± SEMs. Statistical analysis was performed using GraphPad Prism (GraphPad Software). Significance was assessed using the Mann-Whitney *U* test, with *P* < 0.05 considered statistically significant.

## RESULTS

### Liver oxysterol levels in patients with NASH

We acquired liver biopsies from 13 patients during bariatric surgery and control liver samples without peripheral liver pathology findings from 4 patients undergoing partial liver resection. Histopathological analysis revealed NASH in nine bariatric patients and healthy liver tissue in four bariatric and the four remaining patients. NASH patients had a higher BMI and higher C-reactive protein, total cholesterol, ApoA1, and ApoB compared with controls (**Table 1**, supplemental Table S1).

**TABLE 1.** Characteristics of patients with NASH and controls

Variable	NASH (*n* = 9)	Control*^a^* (*n* = 8)	*P*
Females [*n* (%)]	5 (55)	7 (87)	0.294
Age at sample, years [median (IQR)]	35.1 (28.2–38.3)	47.2 (36.6–56.6)	0.059
BMI, kg/m^2^ [median (IQR)]	45.4 (44.3–48.8)	31.2 (21.1–36.9)	0.0003
AST,*^b^* U/l [median (IQR)]	28 (25–49)	29 (21–36)	0.299
ALT,*^b^* U/l [median (IQR)]	35 (27–65)	21.5 (16.2–34.2)	0.102
NAS [*n* (%)]			
0	0 (0)	8 (100)	
1	0 (0)	0 (0)	
2	0 (0)	0 (0)	
3	3 (33.3)	0 (0)	
4	3 (33.3)	0 (0)	
5	3 (33.3)	0 (0)	
Fibrosis	5 (55.5)	0 (0)	

Statistical comparisons were carried out with the use of the Mann-Whitney *U* test and Fisher’s exact test.

a The control group includes four bariatric patients undergoing liver biopsy during surgery without NASH and without liver steatosis and patients undergoing partial liver resection due to liver metastasis of (one each) pancreatic carcinoma, rectum carcinoma, and gall bladder carcinoma as well as *Ecchinococcus*.

b Normal: <35 females and <50 males.

**Fig. 1.**
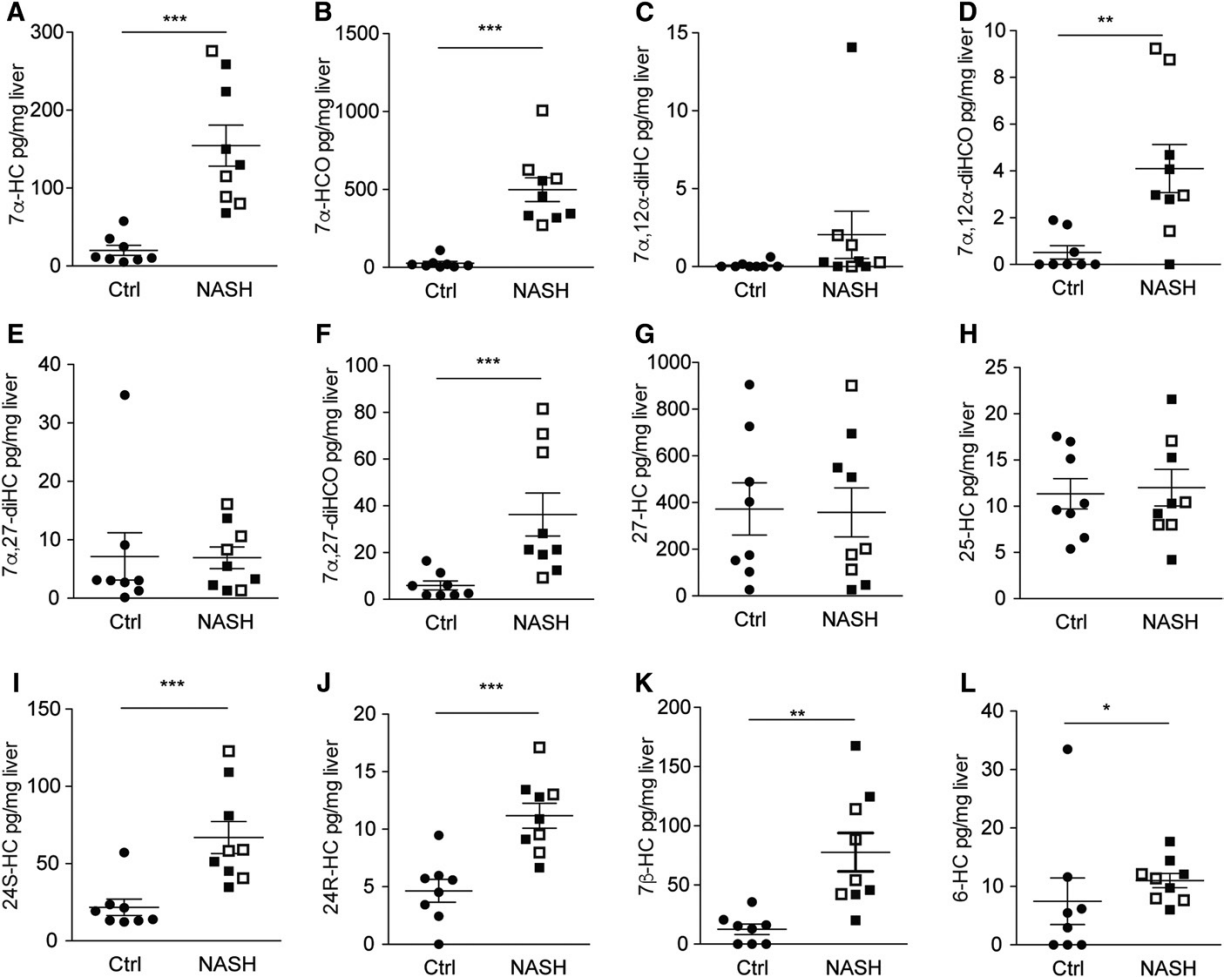
Liver oxysterol levels in patients with NASH versus controls**.** Levels of various oxysterols were determined in human liver samples from nine patients with NASH (black squares: with fibrosis; white squares: no fibrosis) and eight controls. Statistical analysis: Mann-Whitney ***U*** test. ***P < 0.001, **P < 0.01, and *P < 0.05.

In comparing free oxysterol levels in bariatric patients with NASH with controls without NASH, we found higher levels of 7α-HC, 7α-HCO, 7α,27-diHCO, and 7α,12α-diHCO (**Scheme 1A**, **Fig. 1**). Further, levels of 24S-HC, 24R-HC, and 7β-HC were increased in NASH. In contrast, concentrations of 25-HC and 27-HC did not differ between individuals with and without liver inflammation. Levels of 7α-HC, 7α-HCO, 7α,27-diHC, 24S-HC, 24R-HC, and 7β-HC strongly correlated with disease activity, as assessed by the quantification of liver inflammation using the NAFLD activity score (NAS) (**Table 2**), confirming the possible association of increased oxysterols with NASH.

Scheme 1. Chemical structure and synthesis of oxysterols. Red arrows indicate higher levels found in NASH patients compared with controls without NASH.
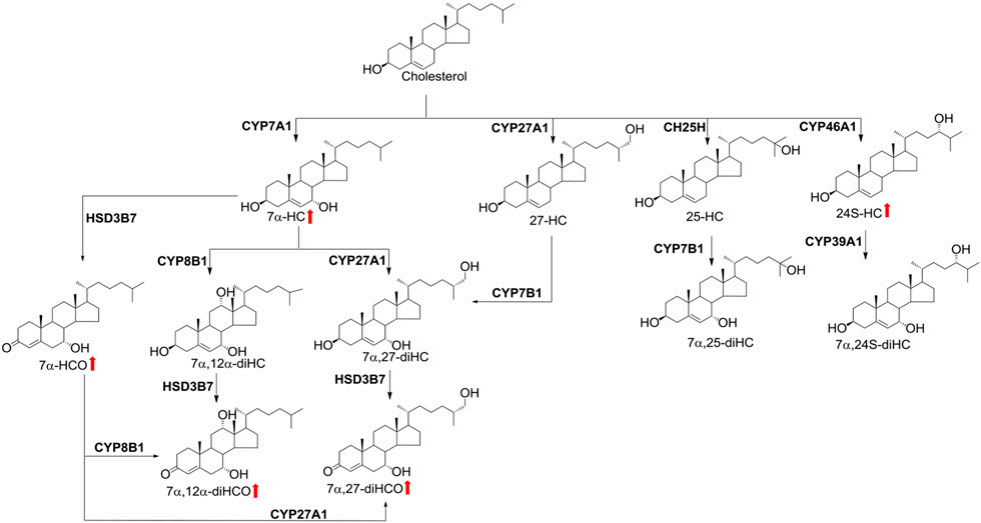


**TABLE 2.** Correlation of oxysterol levels with NASH disease activity

Measured Oxysterol (pg/mg liver)	Humans			Mice		
	Control (*n* = 8) [median (min − max)]	NASH (*n* = 9) [median (min − max)]	Correlation with NAS: *r*, *p*	STD (*n* = 8) [median (min − max)]	HFD (*n* = 10) [median (min − max)]	Correlation with NAS: *r*, *p*
7α-HCO	14.82 (2.69–108.79)	457.20 (271.19–1006.33)	**0.744, 0.0006**	238.36 (59.67–1709.92)	709.85 (245.31–1709.92)	0.184, 0.4651
7α,27-diHCO	4.20 (1.67–16.46)	21.29 (9.27–81.52)	**0.685, 0.0024**	60.26 (30.18–106.49)	49.57 (29.12–106.49)	−0.212, 0.3983
7α,12α-diHCO	0 (0–1.90)	2.97 (0–9.24)	0.474, 0.0545	9.24 (2.12–22.41)	7.70 (2.77–22.41)	−0.280, 0.2602
7α-HC	10.93 (5.00–57.66)	129.93 (68.23–276.22)	**0.877, <0.0001**	87.01 (18.37–552.89)	76.34 (0–552.89)	−0.268, 0.2815
7α,27-diHC	3.06 (0.15-–34.80)	5.48 (1.33–16.10)	0.098, 0.7087	1.91 (0–32.81)	5.16 (0–32.81)	−0.019, 0.9414
7α,12α-diHC	0 (0–0.62)	0.28 (0–14.07)	0.373, 0.1404	0 (0–2.90)	0.33 (0–2.90)	**0.500, 0.0345**
7β-HC	14.10 (0–35.64)	54.15 (20.11–167.52)	**0.756, 0.0004**	84.03 (47.88–1099.57)	314.98 (100.54–1099.57)	**0.493, 0.0375**
6-HC	4.20 (0–33.47)	11.38 (6.00–17.67)	0.475, 0.0542	14.99 (4.45–481.55)	72.26 (29.28–481.55)	0.184, 0.4651
24S-HC	16.6 (12.3–57.2)	58.32 (34.85–122.83)	**0.757, 0.0004**	3.67 (1.05–18.87)	12.43 (4.42–18.87)	**0.713, 0.0009**
24R-HC	5.06 (0–9.47)	10.89 (6.65–17.10)	**0.779, 0.0002**	15.35 (7.17–99.51)	64.44 (42.45–99.51)	**0.817, <0.0001**
25-HC	9.95 (5.39–17.56)	10.30 (4.21–21.58)	0.086, 0.7425	15.46 (4.00–61.19)	40.05 (13.00–61.19)	**0.476, 0.0459**
27-HC	288.92 (25.89–905.65)	202 (25.93–900.90)	0.053, 0.8384	83.03 (4.32–725.84)	226.40 (34.15–725.84)	0.329, 0.1827

Oxysterols levels (pg /mg liver tissue) of human and murine liver samples and correlations of the respective oxysterol concentration with histological disease activity (NAS) are indicated. Significant correlations are shown in bold. Spearman’s rank-order correlation was used for statistical analysis. 6-HC was derived from cholestan-3β,5α,6β-triol during sample preparation.

Levels of liver free cholesterol were slightly higher in NASH livers but not significantly different from the control (supplemental Fig. S1), suggesting that increased levels of free oxysterols are not simply due to the general effects of NASH on sterol composition. In fact, when oxysterol levels were normalized to cholesterol concentration, all significant differences for oxysterols remained in place, even though with slightly lower significance (supplemental Fig. S2).

### A murine long-term feeding model of NASH with increased body weight, liver steatosis, and inflammation

**Fig. 2.**
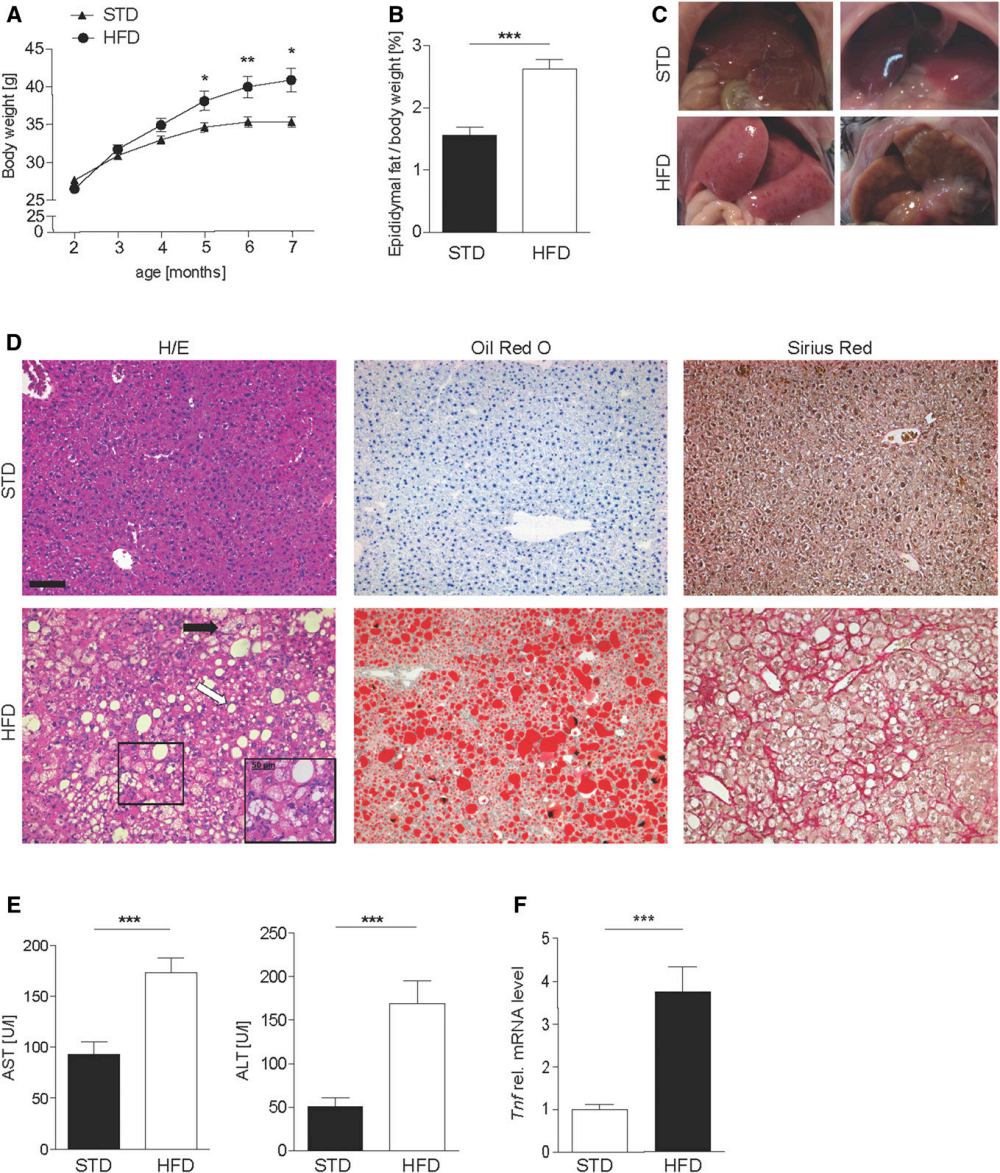
Increased body weight and liver inflammation in mice fed an HFD for 20 weeks. A: Weight development in male HFD and STD C57BL/6 mice. B: Epididymal fat weight as a percentage of total body weight after 20-week HFD feeding in C57BL/6 mice. C: Macroscopic aspect of livers from STD and HFD C57BL/6 mice. D: Representative H/E (left), Sirius Red (middle), and Oil Red O staining (right) of STD (upper panel) and HFD (bottom panel) wild-type mice, illustrating steatosis (white arrow), necroinflammation (inserts with magnification), cellular hypertrophy (black arrow), fat accumulation (middle panel), and collagen deposition (right panel). Scale bar: 100 µm. E: Serum liver function tests (AST and ALT) in HFD and STD mice. F: Liver tissue was analyzed for mRNA expression of *Tnf,* normalized to *Hprt*. Statistical analysis: Mann-Whitney *U* test, *n* ≥ 9. ***P < 0.001, **P < 0.01, and *P < 0.05.

**Fig. 3.**
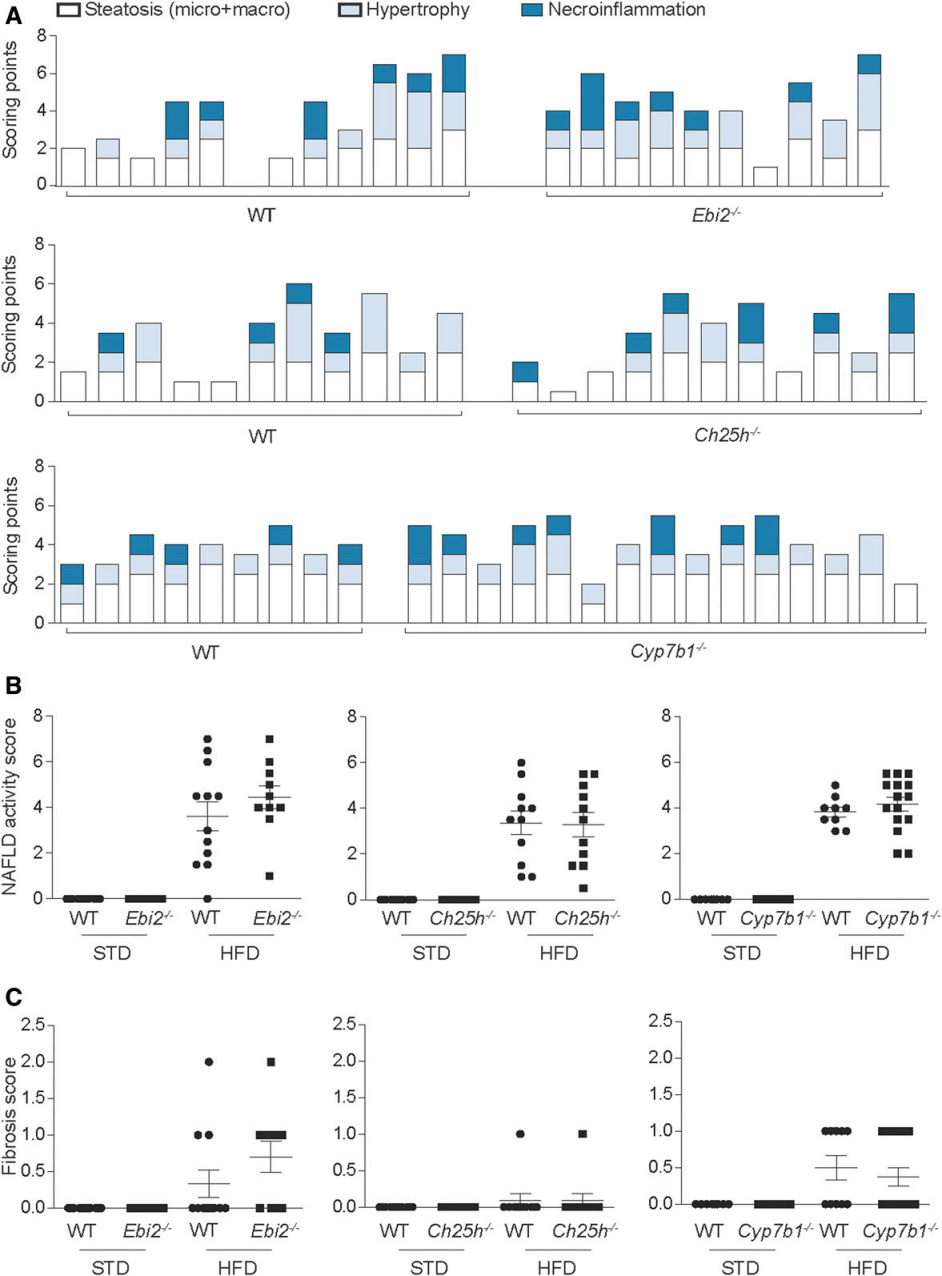
EBI2, CH25H, and CYP7B1 are not essential for the induction of NASH by HFD. A: Quantification of liver inflammation by the NAS of *Ebi2^−/−^* (upper panel), *Ch25h^−/−^* (middle panel), and *Cyp7b1^−/−^* (bottom panel). Each column represents one individual mouse. White fractions of bars represent steatosis scoring (including both micro- and macrosteatosis), light blue fractions represent cellular hypertrophy, and dark blue fractions represent necroinflammation scoring. Quantification of (B) NAS and (C) fibrosis score in STD and HFD knockout mice and the respective littermate controls. Each dot represents one mouse, and wild-type mice are shown together with their respective littermate controls; wt vs. *Ebi2^−/−^*: *n* = 12 + 10, wt vs. *Ch25h^−/−^*: *n* = 11 + 11, and wt vs. *Cyp7b1^−/−^*: *n* = 9 + 15.

To study the role of oxysterols in NAFLD/NASH in more detail, we established a murine feeding model in C57BL/6 mice. Eight-week-old male mice were fed for 20 weeks with a high-fat, high-cholesterol diet (75% fat, 14% carbohydrates, 11% proteins, 1.25% w/w cholesterol) and high-fructose corn-syrup equivalent (55% fructose and 45% glucose) in the drinking water (42) (HFD) or with standard chow and normal drinking water as the control (STD). We observed a steady increase in body weight (**Fig. 2A**), resulting in a significantly higher weight in HFD mice at the end of the experiment (HFD: 40.7 ± 6.2 g; STD: 35.2 ± 2.8 g; *P* = 0.01). Epididymal fat of HFD mice was also increased, indicating changes in fat distribution upon long-term HFD feeding (Fig. 2B). Macroscopically, livers were enlarged and pale (for representative images compare Fig. 2C). Histological analysis of H/E-stained liver sections of HFD mice revealed steatosis in 31 of 32 (97%; white arrow in Fig. 2D, left panel), cellular hypertrophy in 26 of 32 (81%; black arrow), and necroinflammation in 15 of 32 (47%; magnified in the inset). Steatosis was also apparent with Oil Red O staining, which showed a pronounced accumulation of fat droplets in HFD mice (Fig. 2D, right panel). Scores for steatosis, cellular hypertrophy, and necroinflammation were assigned according to a general NAFLD scoring system for rodent models (44), which is an adaption of the classification of Kleiner et al. (45). NASH is histologically defined as the combination of each of the following three key elements: *i*) steatosis, *ii*) cellular hypertrophy or ballooning, and *iii*) the presence of necroinflammation. Overall, 15 of 32 (47%) C57BL/6 mice developed NASH (**Fig. 3**, **Table 3**).

**TABLE 3.** Histological scores of the HFD-fed knockout mice and the respective littermate controls

		Wild-Type: *Ebi2^+/+^* (*n* = 12)	*Ebi2^−/−^* (*n* = 10)	Wild-Type: *Ch25h^+/+^* (*n* = 11)	*Ch25h^−/−^* (*n* = 11)	Wild-Type: *Cyp7b1^+/+^* (*n* = 9)	*Cyp7b1^−/−^* (*n* = 15)*^-^*
Histological Feature	Score/Code	*n* (%)					
Steatosis grade	<1	1 (8)	0	0	0	0	0
	≥1 to <2	6 (50)	2 (20)	6 (54.5)	6 (55)	1 (11)	1 (7)
	≥2 to <3	4 (33)	7 (70)	5 (45.5)	5 (45)	6 (67)	11 (73)
	3	1 (8)	1 (10)	0	0	2 (22)	3 (20)
Lobular inflammation	0	6 (42)	4 (40)	7 (63.6)	5 (45)	4 (44)	8 (53)
	1	4 (33)	5 (50)	4 (36.4)	4 (36)	5 (56)	4 (27)
	2	2 (25)	0	0	2 (18)	0	3 (20)
	3	0	1 (10)	0	0	0	0
Cellular hypertrophy	0	3 (25)	2 (20)	3 (27.3)	4 (36)	0	1 (7)
	1	5 (42)	3 (30)	4 (36.4)	5 (45)	9 (100)	11 (73)
	2	2 (17)	4 (40)	2 (18.2)	2 (18)	0	3 (20)
	3	2 (17)	1 (10)	2 (18.2)	0	0	0
Fibrosis stage	0	9 (75)	4 (40)	10 (90.9)	10 (91)	5 (56)	10 (67)
	1-1a	1 (8)	4 (40)	1 (9.1)	1 (9)	4 (44)	3 (20)
	1b	1 (8)	1 (10)	0	0	0	2 (13)
	1c	0	0	0	0	0	0
	≥2	1 (8)	1 (10)	0	0	0	0

Normal liver histology was observed for all control mice without evidence of steatosis, hypertrophy, or necroinflammation, resulting in a NAS of 0 for all mice tested (Fig. 3A, B).

Fibrosis, evaluated on Sirius Red-stained sections and quantified according to Kleiner et al. (45), was present in 6 of 32 mice (19%) with a grade of 1-1a; only 2 mice (6%) developed a higher degree of fibrosis (Fig. 2D, middle panel; Fig. 3C; supplemental Table S2).

Liver damage in HFD mice was confirmed by significantly elevated AST and ALT serum levels compared with the STD group (Fig. 2E). These changes were accompanied by enhanced expression of *Tnf* mRNAs in liver tissue (Fig. 2F), pointing to ongoing liver inflammation. Taken together, all histological, biochemical, and immunological hallmarks for NASH were present in approximately 50% of the mice fed a long-term HFD in this study, confirming this model as a valid physiological murine model for the development of NAFLD and NASH.

### EBI2, CH25H, and CYP7B1 are not essential for HFD-induced NASH

To clarify the role of the EBI2-7α,25-diHC system in hepatic inflammation, we analyzed H/E-stained liver sections of HFD-fed knockout mice of key players of oxysterol production and signaling (*Ebi2^−/−^*, *Ch25h^−/−^*, and *Cyp7b1^−/−^*) and their respective wild-type littermate controls. Histological hallmarks of NASH (steatosis, cellular hypertrophy, and necroinflammation) were detected in most mice after 20 weeks of HFD feeding; however, no differences were noted when knockout mice (*Ebi2^−/−^*, *Ch25h^−/−^*, and *Cyp7b1^−/−^*) and their respective littermate wild-type controls were compared (Fig. 3A, B). Fibrosis was also indistinguishable between wild-type and knockout mice (Fig. 3C). As expected, no liver pathology was observed in any of these knockout mice upon STD feeding.

**Fig. 4.**
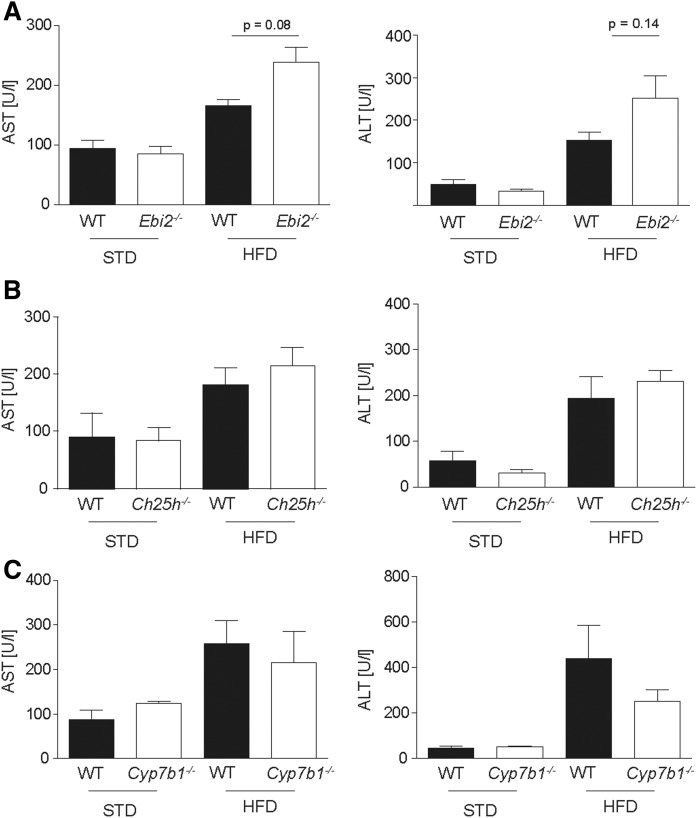
HFD increases liver function tests in mice independent of EBI2, CH25H, and CYP7B1 gene function. Quantification of serum AST and ALT in STD and HFD (A) *Ebi2^−/−^*, (B) *Ch25h^−/−^*, and (C) *Cyp7b1^−/−^* mice and the respective littermate wild-type controls (*n*_STD_ = 2–8; *n*_HFD_ ≥ 9). Statistical analysis: Mann-Whitney *U* test.

**Fig. 5.**
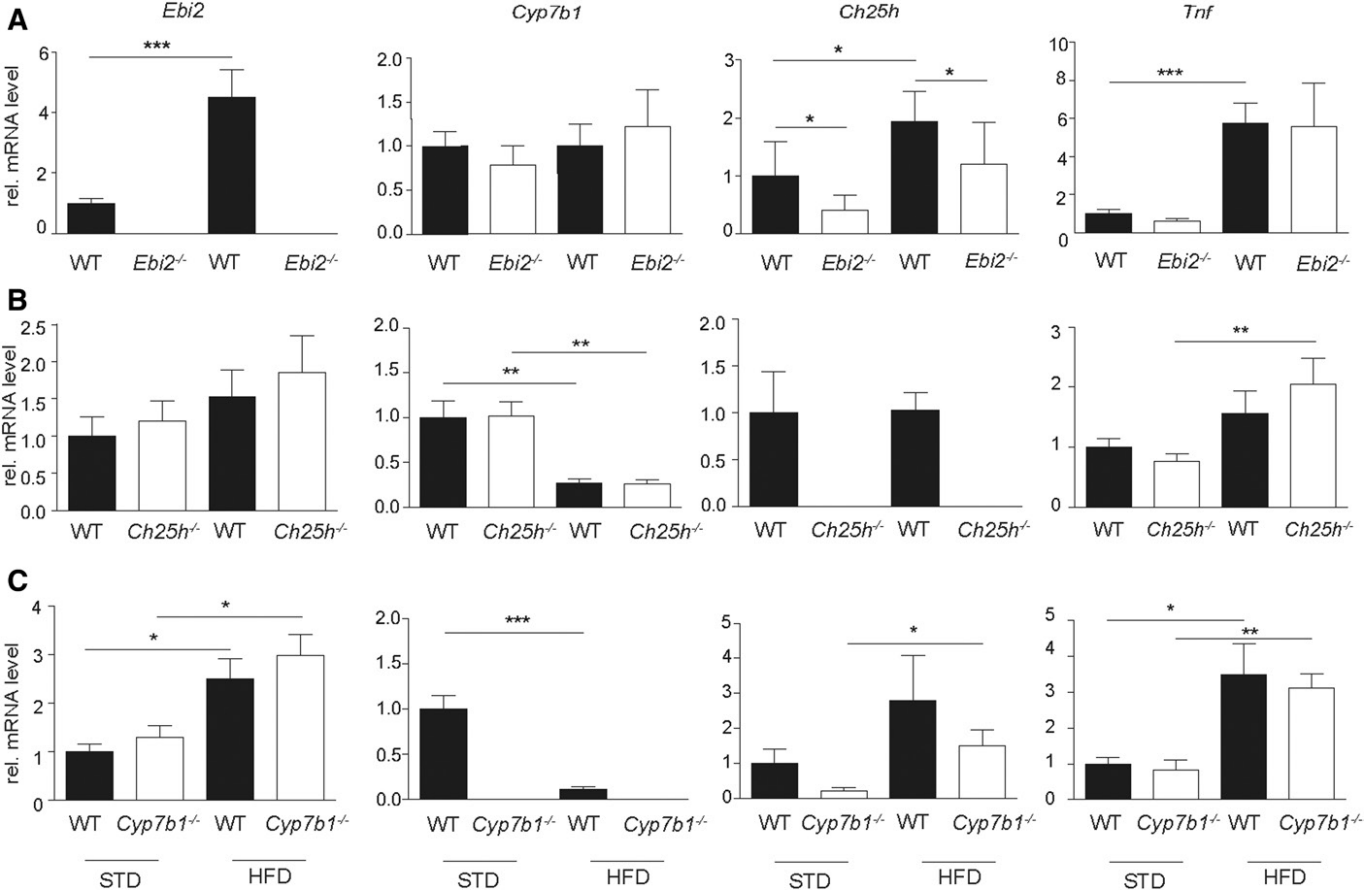
HFD changes the expression levels of genes involved in oxysterol metabolism and increases the expression of *Tnf* independent from EBI2, CH25H, and CYP7B1 gene function. Quantification of liver tissue mRNA level of *Ebi2*, *Cyp7b1*, *Ch25h*, and *Tnf* in STD and HFD (A) *Ebi2^−/−^*, (B) *Ch25h^−/−^*, and (C) *Cyp7b1^−/−^* mice and the respective littermate wild-type controls (*n*_STD_ ≥ 5; *n*_HFD_ ≥ 9). Statistical analysis: Mann-Whitney *U* test. ***P < 0.001, **P < 0.01, and *P < 0.05.

Liver inflammation was confirmed by elevated serological markers for liver inflammation, including AST and ALT upon HFD feeding, but no effects of EBI2, CH25H, and CYP7B1 knockouts were detected (**Fig. 4**). In line with these observations, the liver tissue mRNA expression level of *Tnf* as a marker for inflammation was increased upon HFD feeding, but no genotype-related differences were detected (**Fig. 5**).

Additionally, mRNA analysis of the key players of the EBI2-7α,25-diHC system in wild-type mice revealed significant increases of *Ebi2* and *Ch25h* levels and a significant downregulation of *Cyp7b1* upon HFD feeding when pooling all wild-type mice of the three independent experiments (supplemental Fig. S3). However, when the different knockout mice were compared with their respective wild-type littermate controls, the differences in *Ebi2* and *Cyp7b1* expression were not significant. Surprisingly, expression levels of *Ch25h* were decreased in *Ebi2^−/−^* knockout mice compared with wild-type littermate controls for STD as well as after HFD feeding (Fig. 5).

To test for the effects of *Ebi2*, *Ch25h*, and *Cyp7b1* at an initial stage in NASH pathogenesis a different time point was tested. After 10 weeks of HFD feeding most mice developed steatosis (33 of 34; 97%) and cellular hypertrophy (28 of 34; 83.2%), but no necroinflammation was detectable at this time point (supplemental Fig. S4). Similar to the results for the 20-week HFD feeding, steatosis and cellular hypertrophy scores were indistinguishable between the wild-type and knockout mice with a deficiency in the EBI2-7α,25-diHC system.

### Recruitment of immune cells into the inflamed liver did not differ in mice with a deficient EBI2-7α,25-diHC system

Intrahepatic mononuclear cells were isolated from livers after 20-week HFD or STD feeding, and flow cytometry analysis was performed to test for the differential recruitment of immune cells in mice with a dysfunctional EBI2-7α,25-diHC system. However, no differences were detected in major immune cell populations, including B-cells, CD4^+^ T-helper cells, and CD8^+^ cytotoxic T-cells (supplemental Fig. S5). Only very low numbers of dendritic cells and macrophages were present in the liver of STD- and HFD-fed mice, making quantification inappropriate.

### Increased liver oxysterol and cholesterol levels upon HFD feeding

**Fig. 6.**
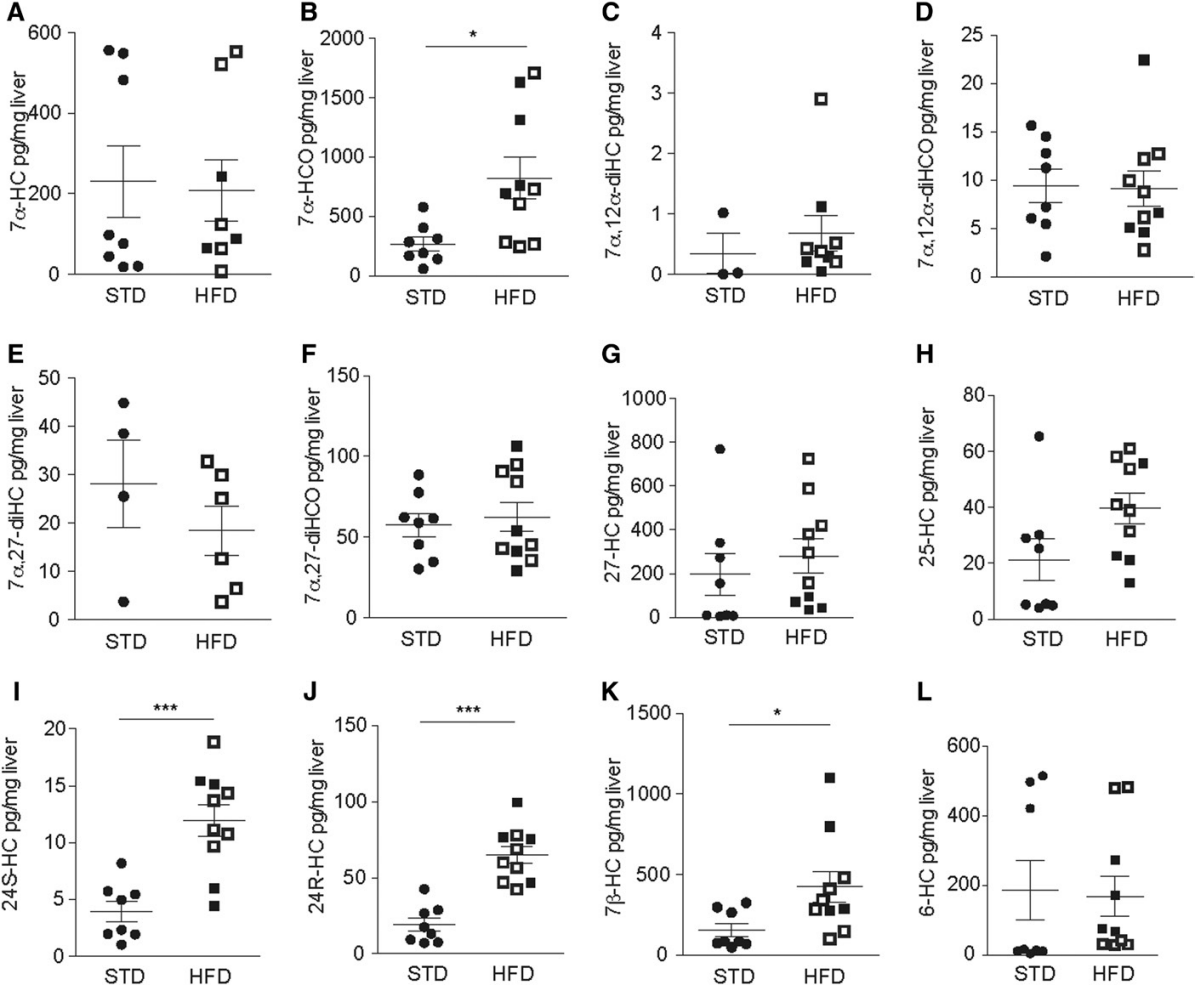
Liver oxysterol levels in a murine model of NAFLD/NASH. Eight-week-old male C57BL/6 mice were fed an HFD or STD for 20 weeks. Levels of the indicated oxysterol in the liver tissue of HFD (black squares: NASH; white squares: NAFLD) and STD controls were measured by LC-MS. Statistical analysis: Mann-Whitney *U* test (*n*_STD_ = 8, ≥3 valid data points; *n*_HFD_ = 10, ≥5 valid data points). ***P < 0.001, **P < 0.01, and *P < 0.05.

Free oxysterols and cholesterol measurements by LC-MS in mouse livers revealed elevated levels for some oxysterols in HFD-fed C57BL/6 mice (**Fig. 6**, supplemental Table S3).

Similar to humans, levels of 7α-HCO were significantly elevated upon HFD feeding. However, the difference was smaller compared with human samples, and HFD feeding did not alter 7α,27-diHCO and 7α,12α-diHCO levels. As in humans, 24-hydroxylated oxysterols (24S-HC, 24R-HC) were significantly increased upon HFD feeding. In addition, 22R-HC, 20R,22R-diHC, and 20R,22R-diHCO, which were not detectable in human livers, were also significantly increased (supplemental Fig. S6). HFD feeding also significantly increased cholesterol levels (supplemental Fig. S6A). After normalizing for liver cholesterol levels, the difference in 7α-HCO concentrations was no longer significant (supplemental Fig. S7).

## DISCUSSION

In this study, we performed a detailed analysis regarding the role of oxysterols and the oxysterol-EBI2 axis in NASH using human samples and murine samples from a feeding model of NASH. We demonstrated a NAFLD/NASH-dependent increase in free oxysterols in human livers and in mice. Detailed experiments in a murine feeding model of NASH demonstrated an upregulation of *Ebi2* and *Ch25h* and a downregulation of *Cyp7b1* (supplemental Fig. S3). However, knockouts of EBI2 and the oxysterol-producing enzymes *Ch25h* and *Cyp7b1* did not show significant differences in NAFLD disease activity, excluding an essential role of these genes, at least in this murine model of NASH.

The level of 7α-HCO is normally regarded as a marker for bile acid synthesis. It has been reported that the serum level of 7α-HCO is significantly increased in patients with NASH (46–48). In another study, it was shown that the mRNA expression of CYP7A1, the rate-limiting enzyme in bile acid synthesis, was induced in human NASH livers (49). In partial agreement with a previous study (48), we found that the level of 7α-HC and its downstream metabolites 7α-HCO, 7α,12α-diHCO, and 7α,27-diHCO were significantly higher in NASH livers, which directly confirmed the upregulation of bile acid synthesis associated with NASH. We also found an increase of 24S-HC and 24R-HC in humans and mice with NASH. 24S-HC is mainly produced in the brain by the enzyme cholesterol 24S-hydroxylase (CYP46A1), which is expressed in neurons. After enzymatic conversion, 24S-HC is able to cross the blood-brain barrier and is metabolized in the liver by the enzyme 24-hydroxycholesterol 7-α-hydroxylase (CYP39A1) to 7α,24S-diHC and then to bile acids. To the best of our knowledge, the 24-HC epimers have, so far, not been implicated in NASH. The mechanism of 24-HC accumulation in NASH remains unclear; the most likely explanation for 24S-HC accumulation is decreased downstream metabolism. The origin of 24R-HC is not known, and it is seldom analyzed in MS studies. The level of 7α,24S-diHC is below its detection limit (<5 pg/mg liver), but in future studies we will investigate whether CYP39A1 is downregulated in NASH livers.

In clinical practice, the diagnosis of NASH remains difficult, and reliable biomarkers are lacking. Liver biopsy is considered the gold standard for diagnosing NASH, which remains an invasive procedure and can both over- or underestimate inflammatory activity due to sampling error. The pattern of increased levels of 7α-HC, 7α-HCO, 24S-HC, and 24R-HC suggest that they could be used as markers for NASH. Moreover, these oxysterols strongly and significantly correlated with the severity of NASH according to the NAFLD disease severity score. A future study will check whether these changes are reflected in plasma. A panel of 20 metabolites has been suggested for the distinction of NASH from steatosis in a previous lipidomics study (48).

NAFLD and NASH have been intensively studied in animal models (50). However, the validity of some animal models has been questioned: The use of a methionine- and choline-deficient diet and/or genetic models, including mutations or knockout of key genes for the regulation of eating behavior such as the leptin or leptin receptor gene, can rapidly induce liver inflammation, but these nonphysiological interventions result in the loss of important aspects of NAFLD pathogenesis (51, 52). For our study, we established a high-fat, high-cholesterol, high-fructose diet murine feeding model of NAFLD, which spontaneously progresses to steatohepatitis. As a result, obesity with increased epididymal adiposity, increased AST and ALT serum levels, and upregulated liver expression of the inflammatory marker *Tnf* were induced, reflecting features of the human disease. In our experiments, steatohepatitis developed in approximately 50% of the mice in a time-dependent manner, and a low number of mice even progressed to pericellular fibrosis. Compared with other models the degree of liver injury was intermediate; this, together with the fact that steatohepatitis developed in only half of the mice, should be considered as a strength of our study because it allows an assessment of inhibition as well as stimulation of liver steatosis and inflammation by additional interventions, such as the genetic knockout of *Ebi2*, *Cyp7b1*, or *Ch25h*. HFD feeding models such as ours with high caloric intake, rich in cholesterol, saturated fats, and fructose are time-consuming but adequately reflect the human condition.

Several lines of evidence suggest a broad and general role of oxysterols in NASH pathogenesis (14, 15, 53, 54). However, here we show that loss of the enzymes CH25H and CYP7B1, and thus inhibition of 25-HC and 7α,25-diHC synthesis, did not show any significant differences in NASH activity compared with their wild-type littermate controls, arguing against an essential role of these two enzymes and their immediate products in NASH pathogenesis. However, cholesterol oxidation at position 25 is not limited to CH25H, and the action of other enzymes, including CYP27A1 and CYP3A4, can also lead to 25-HC (55). CYP3A11 in mice and CYP3A4 in humans can introduce a 25-hydroxy group to 7α-HC, providing an alternative route to 7α,25-diHC via CYP7A1 and CYP3A4 in humans or CYP3A11 in mice (56). Therefore, our results do not completely exclude a role of 25-HC in NASH pathogenesis.

A previous study reported that the hematopoietic depletion of CYP27A1 and thus the inhibition of 27-HC synthesis in another murine NASH model with knockout of low-density lipoprotein receptor (*Ldlr^−/−^*) and an HFD feeding for 12 weeks resulted in stronger NASH activity upon CYP27A1 depletion compared with wild-type controls. In line with these data, 27-HC applied subcutaneously substantially decreased liver inflammation (15). Furthermore, hematopoietic overexpression of CYP27A1 in the same mouse model was able to rescue the diet-induced liver inflammation (54). Due to differences of the animal models and the experimental approach, a direct comparison to our results is not possible. We stress the importance of using littermate wild-type controls given the recognized crucial effect of the microbiota during the development of NAFL and NASH (57–61), which was rigorously done in all of our experiments. Further experiments will clarify whether 27-HC [as suggested by Bieghs et al. (15)] is a critical player for the induction and progression of NASH.

24S, 25-HC, 27-HC and other side-chain oxysterols can retain sterol response element-binding proteins on the cytoplasmic surface of the endoplasmic reticulum and prevent their transcriptional activity (62). This results in reduced cholesterol synthesis and suppressive effects on the immune system (63). Additionally, side-chain oxysterols are agonists of the liver X receptors (LXRs) (55). The effects of LXR agonists on NASH are complex. On the one hand, LXR activation has broad anti-inflammatory effects (64, 65); on the other hand, LXR activation would increase endogenous lipogenesis, thus promoting liver steatosis (66). Due to this complexity, experiments in our study neither prove nor exclude a general role of oxysterols through the LXR or SREBP pathway in NAFLD and NASH.

EBI2 was shown to be crucial for the migration of B-, T-, and dendritic cells (30, 31, 33, 67, 68). EBI2 was shown to mediate a fast antibody response (67) and was recently shown to play a significant role in the migration of T-cells in multiple sclerosis (35, 69), the migration of osteoclasts precursors in osteoporosis (70), and the activity of innate lymphoid cells in colitis (38). We report no effect of EBI2, CH25H, or CYP7B1 knockouts in the complex mechanisms of steatosis and inflammation during NASH pathogenesis, making essential effects of EBI2 or its ligand 7α,25-diHC in NAFLD/NASH highly unlikely. Increased EBI2 enzyme levels in NASH livers (Fig. 5) might reflect an invasion of liver tissue with EBI2-expressing immune cells. However, only a minority of mice developed liver fibrosis. While no obvious effects on the limited number of animals with all knockout could be detected, further experiments in a liver fibrosis model might be warranted to show or exclude the effects of EBI2 on liver fibrosis.

An interesting observation in mice fed a HFD is the increased abundance in the liver of the oxysterols 22R-HC and 20R,22R-diHC; these are the first two metabolites in the biosynthesis of steroid hormones from cholesterol in a reaction catalyzed by CYP11A1 (cholesterol side-chain cleavage enzyme), ultimately giving pregnenolone (71).

The clinical diagnosis of NASH remains a diagnostic challenge. The gold standard liver biopsy is an invasive procedure, and complications, including clinical significant bleeding, can occur. In addition, the degree of NASH could be over- or underestimated by liver biopsy due to sampling error (72). Thus, a noninvasive and reliable diagnostic tool for NASH is an unmet medical need, and future studies must clarify whether 24-HC in liver tissue or (preferably) plasma can be used as a diagnostic marker for NASH.

## Supplementary Material

Supplemental Data
