# Supplementary material for: Elevated oxysterol levels in human and mouse livers reflect nonalcoholic steatohepatitis
Source: J Lipid Res. 2019 May 21;60(7):1270–83. doi: 10.1194/jlr.M093229 (PMC6602130; doi:10.1194/jlr.M093229)
Supplement: Supplemental Data [file supp_60_7_1270__index.html]

Elevated oxysterol levels in human and mouse livers reflect non-alcoholic steatohepatitis — Elevated oxysterol levels in human and mouse livers reflect nonalcoholic steatohepatitis — Supplemental Data 

# Elevated oxysterol levels in human and mouse livers reflect nonalcoholic steatohepatitis

## Supplemental Data

- Supplemental Figures (.pdf, 1.0 MB) - Supplemental figures and tables
