# Supplementary material for: Elevated oxysterol levels in human and mouse livers reflect nonalcoholic steatohepatitis
Source: J Lipid Res. 2019 May 21;60(7):1270–83. doi: 10.1194/jlr.M093229 (PMC6602130; doi:10.1194/jlr.M093229)
Supplement: Supplemental Data [file 10.1194_M093229_jlr.M093229-1.pdf]

Supplemental Material

Variable (median, IQR)	NASH (n=9)	Control# (n=8)	p-value
CRP (mg/l) *Norm.: < 5*	6 (5.7-6.8)	2 (0.8-3)	**p=0.021**
IL-6 (mg/l)* *Norm.: < 7*	4.1 (3-5.4)	2 (1.75-2.65)	p=0.059
Glucose (mmol/l)* *Norm.: 3.9-5.6*	5.7 (5-7.2)	5.6 (5-5.95)	p=0.45
HbA1c NGSP (%)* *Norm.: 4.4-5.7*	5.5 (5.1-5.8)	5.4 (5.3-5.4)	p=0.76
HbA1c IFCC (mmol/mol)* *Norm.: 25-39*	37 (32-40)	36 (35-36)	p=0.76
Cholesterol (mmol/l)* *Norm.: < 5*	4.4 (4.2-5.1)	3.4 (3.35-3.55)	**p=0.0136**
LDL cholesterol (mmol/l)** *Norm.: < 3*	2.3 (2.2-2.8)	1.9 (1.85-1.95)	p=0.091
HDL cholesterol (mmol/l)* *Norm.: > 1*	1.16 (1.02-1.26)	1.05 (1.04-1.18)	p=1
ApoA1 (g/l)* *Norm.: > 1.25*	1.31 (1.27-1.36)	1.1 (1.09-1.15)	**p=0.018**
ApoB (g/l)* *Norm.: > 1*	0.85 (0.75-0.92)	0.61 (0.59-0.65)	**p=0.0045**
Free fatty acids (µmol/l)* *Norm.: 100-450*	630 (451-909)	598 (566-695)	p=0.864
Bile acids (µmol/l)*** *Norm.: < 8*	10.1 (7.8-12)	11.6 (9.75-12.6)	p=0.982
TGL (mmol/l)**** *Norm.: < 2*	1.76 (1.58-1.84)	0.82 (0.65-1.63)	p=0.224
Bilrubin (µmol/l) *Norm.: < 21*	7 (6-9)	7 (6-9)	p=0.98
INR	1.1 (1.1-1.1)	1.1 (1-1.2)	p=1
Thrombocytes	261 (193-307)	248 (218-277)	p=0.62

Supplemental Table S1: Biochemical characteristics of patients with NASH and controls. Statistical comparison: Mann-Whitney U test, Fisher's exact test. Nominally significant values are indicated in bold. IQR: interquartile range; CRP: C-reactive protein, IL-6: interleukin 6, HbA1c: haemoglobin A1c, LDL: low-density lipoprotein, HDL: high-density lipoprotein cholesterol, ApoA1/ B: Apolipoprotein A1/ B, TGL: triglycerides, INR: International normalized ratio.

#the control group includes 4 bariatric patients undergoing liver biopsy during surgery without NASH and without liver steatosis and patients undergoing partial liver resection due to liver metastasis of (one each) pancreatic carcinoma, rectum carcinoma and gall bladder carcinoma as well as Ecchinococcus.
*only three values available for control group.
**only two values available for control group.
***seven values available for control group.
****six values available for control group.

Histological feature	Score/ code	WT (*Ebi2*$^{+/+}$) n=12	*Ebi2*$^{-/-}$ n=10	WT (*Ch25h*$^{+/+}$) n=11	*Ch25h*$^{-/-}$ n=11	WT (*Cyp7b1*$^{+/+}$) n=9	*Cyp7b1*$^{-/-}$ n=15
**Steatosis grade**	**<1**	1 (8%)	0	0	0	0	0
	**≥ 1 < 2**	6 (50%)	2 (20%)	6 (54.5%)	6 (55%)	1 (11%)	1 (7%)
	**≥ 2 < 3**	4 (33%)	7 (70%)	5 (45.5%)	5 (45%)	6 (67%)	11 (73%)
	**3**	1 (8%)	1 (10%)	0	0	2 (22%)	3 (20%)
**Lobular inflammation**	**0**	6 (42%)	4 (40%)	7 (63.6%)	5 (45%)	4 (44%)	8 (53%)
	**1**	4 (33%)	5 (50%)	4 (36.4%)	4 (36%)	5 (56%)	4 (27%)
	**2**	2 (25%)	0	0	2 (18%)	0	3 (20%)
	**3**	0	1 (10%)	0	0	0	0
**Cellular hypertrophy**	**0**	3 (25%)	2 (20%)	3 (27.3%)	4 (36%)	0	1 (7%)
	**1**	5 (42%)	3 (30%)	4 (36.4%)	5 (45%)	9 (100%)	11 (73%)
	**2**	2 (17%)	4 (40%)	2 (18.2%)	2 (18%)	0	3 (20%)
	**3**	2 (17%)	1 (10%)	2 (18.2%)	0	0	0
**Fibrosis stage**	**0**	9 (75%)	4 (40%)	10 (90.9%)	10 (91%)	5 (56%)	10 (67%)
	**1-1a**	1 (8%)	4 (40%)	1 (9.1%)	1 (9%)	4 (44%)	3 (20%)
	**1b**	1 (8%)	1 (10%)	0	0	0	2 (13%)
	**1c**	0	0	0	0	0	0
	**≥ 2**	1 (8%)	1 (10%)	0	0	0	0

Supplemental Table S2: Histological characteristics of mice with NASH in a long-term feeding model. Histological grades and fibrosis grades are indicated.

Name	*Cyp7b1*$^{-/-}$ (pg/mg)
7α-HC	127.5
7α-HCO	504.1
7α,12α-diHC	ND
7α,12α-diHCO	21.0
7α,25-diHC	ND
7α,25-diHCO	ND
7α,27-diHC	ND
7α,27-diHCO	145.6
27-HC	51.6
25-HC	176
24S-HC	15.4
24R-HC	21.9
7β-HC	90.8
6-HC	16.26
22R-HC	1.3
20R,22R-diHC	5.7
3β-hydroxycholest-5-enoic acid	3.4
3β,7α-dihydroxycholest-5-enoic acid	ND
7α-hydroxy-3-oxocholest-4-enoic acid	9.7
cholesterol	1.8 (µg/mg)

Supplemental Table S3: Oxysterol profile of a single *Cyp7b1*$^{-/-}$ mouse liver (n=1). ND, not detected.

A

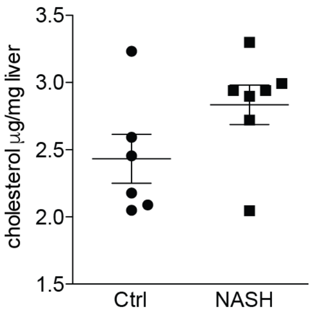

Supplemental Figure S1: Cholesterol levels in human liver tissue in patients with NASH vs. controls. Mann-Whitney U test (no significant differences detected).

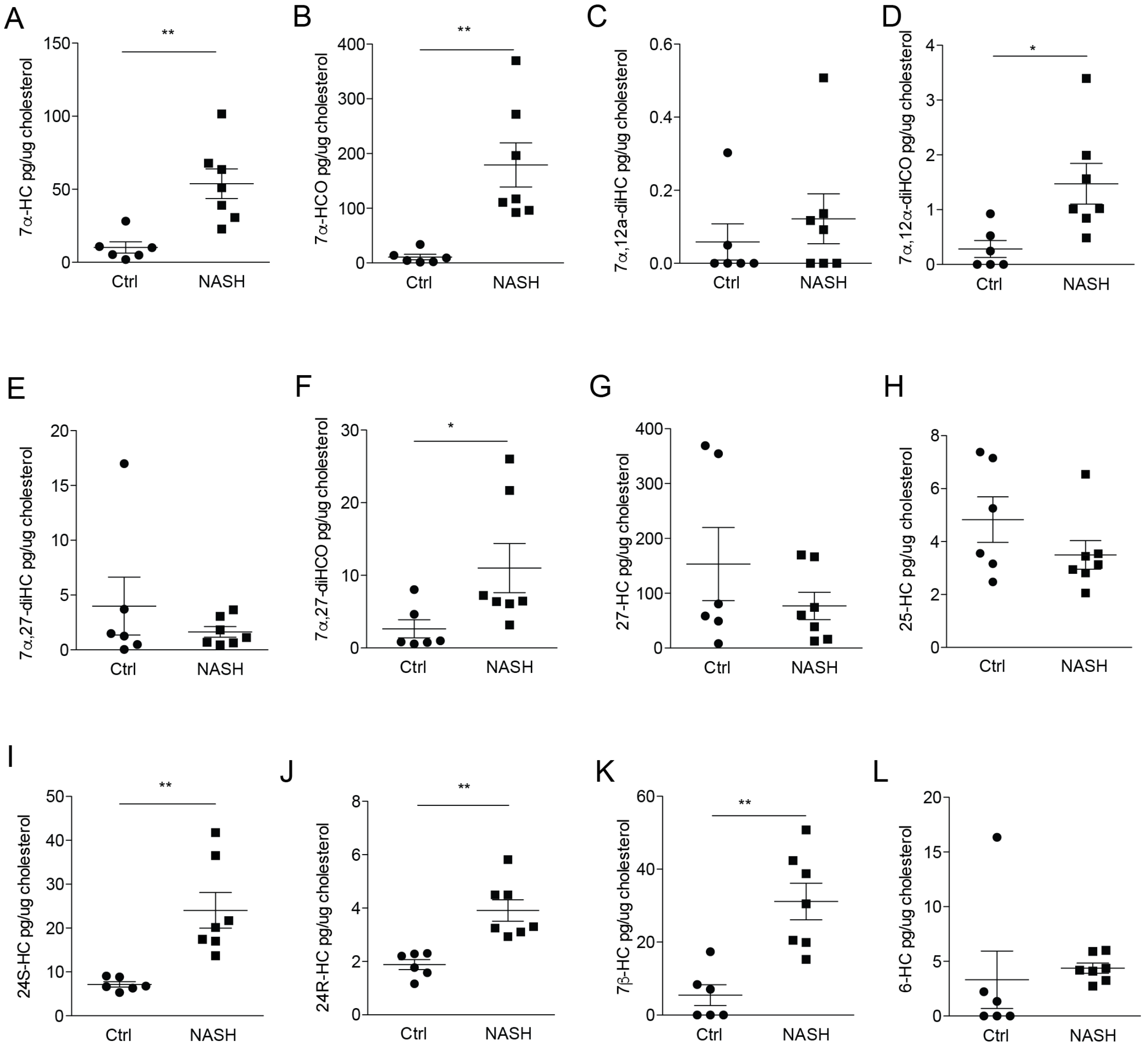

Supplemental Figure S2: Oxysterols levels in human liver tissue in patients with NASH vs. controls normalized to cholesterol levels. Oxysterols levels are indicated as in Figure 1. Oxysterol levels were normalized to the cholesterol concentration within the respective samples (pg/µg cholesterol). Mann-Whitney U test. ***p<0.001, **p<0.01, *p<0.05.

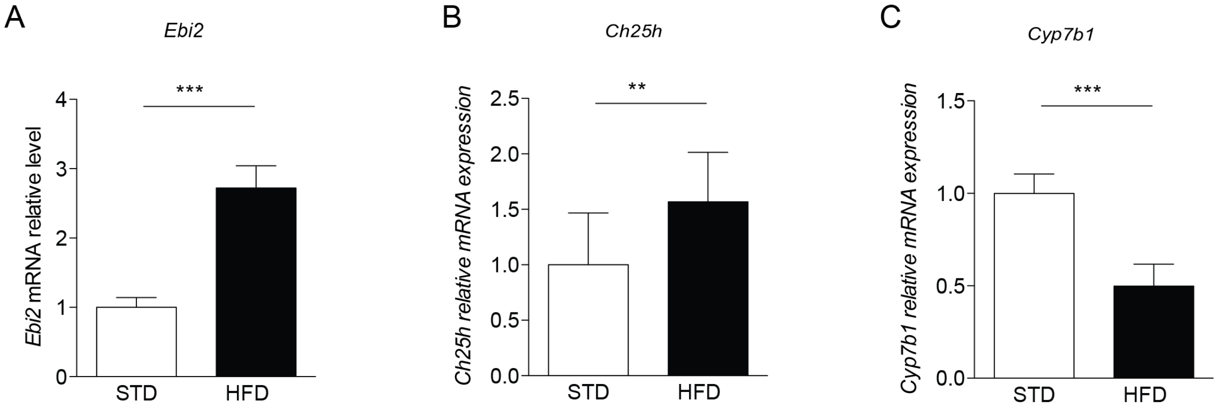

Supplemental Figure S3: HFD changes expression levels of genes involved in the oxysterol metabolism. Quantification of liver tissue mRNA level of (A) *Ebi2*, (B) *Ch25h and (C) Cyp7b1* in STD and HFD fed wildtype mice. ($n_{STD} \geq 20$, $n_{HFD} \geq 28$). Mann-Whitney U test; ***p<0.001, **p<0.01.

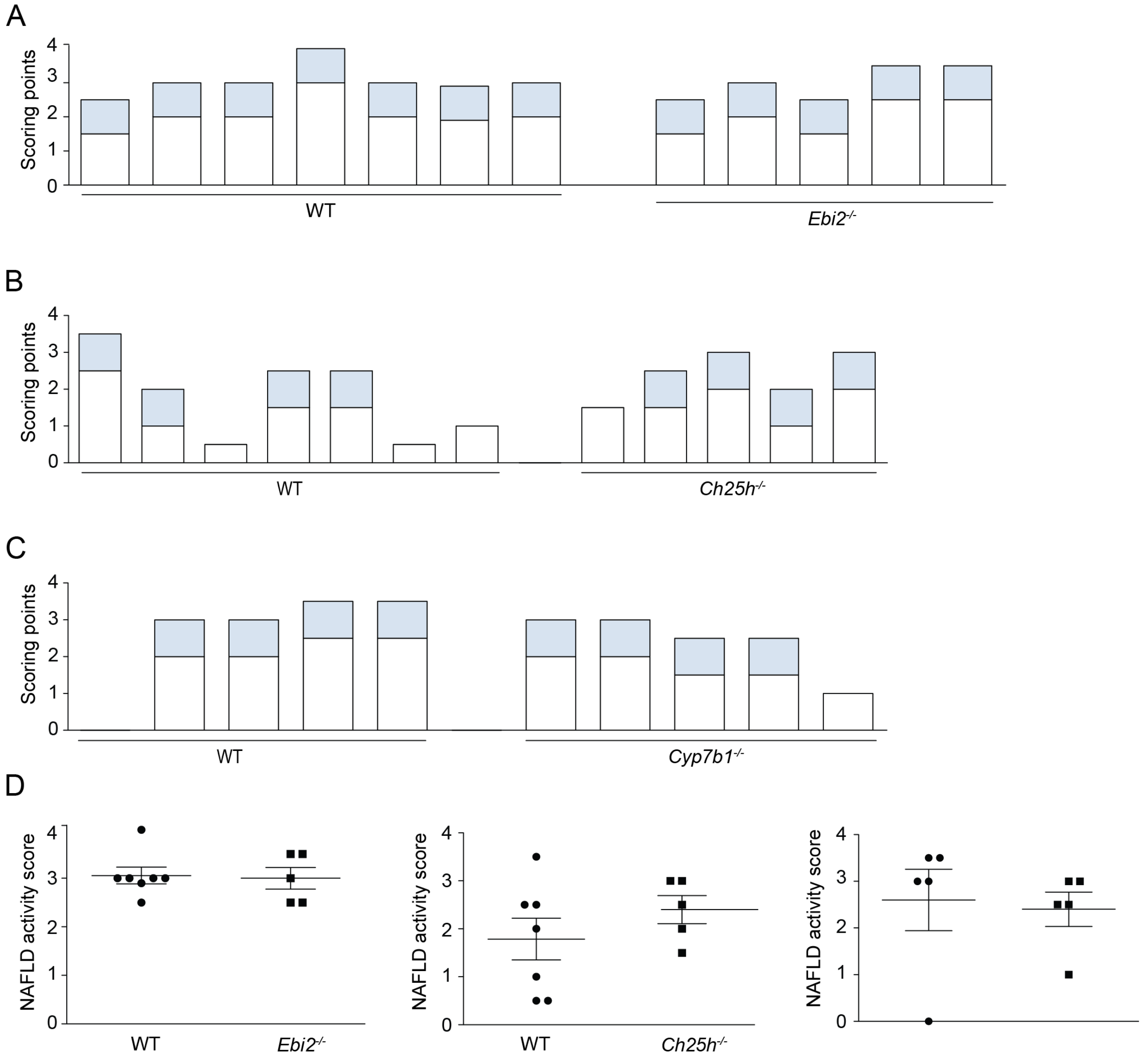

Supplemental Figure S4: EBI2, CH25H and CYP7B1 are not essential for induction of NAFL by 10-weeks HFD feeding. NAFLD activity score of **(A)** *Ebi2*$^{-/-}$, **(B)** *Ch25h*$^{-/-}$ and **(C)** *Cyp7b1*$^{-/-}$. Each column represents one individual mouse. White fractions of bars represent steatosis scoring (including both, micro- and macrosteatosis) and light blue fractions cellular hypertrophy. No necroinflammation was detected. **(D)** Quantification of NAFLD activity score in STD and HFD fed knockout mice and the respective littermate controls. Each dot represents one mouse. Mann-Whitney U test (no significant differences detected).

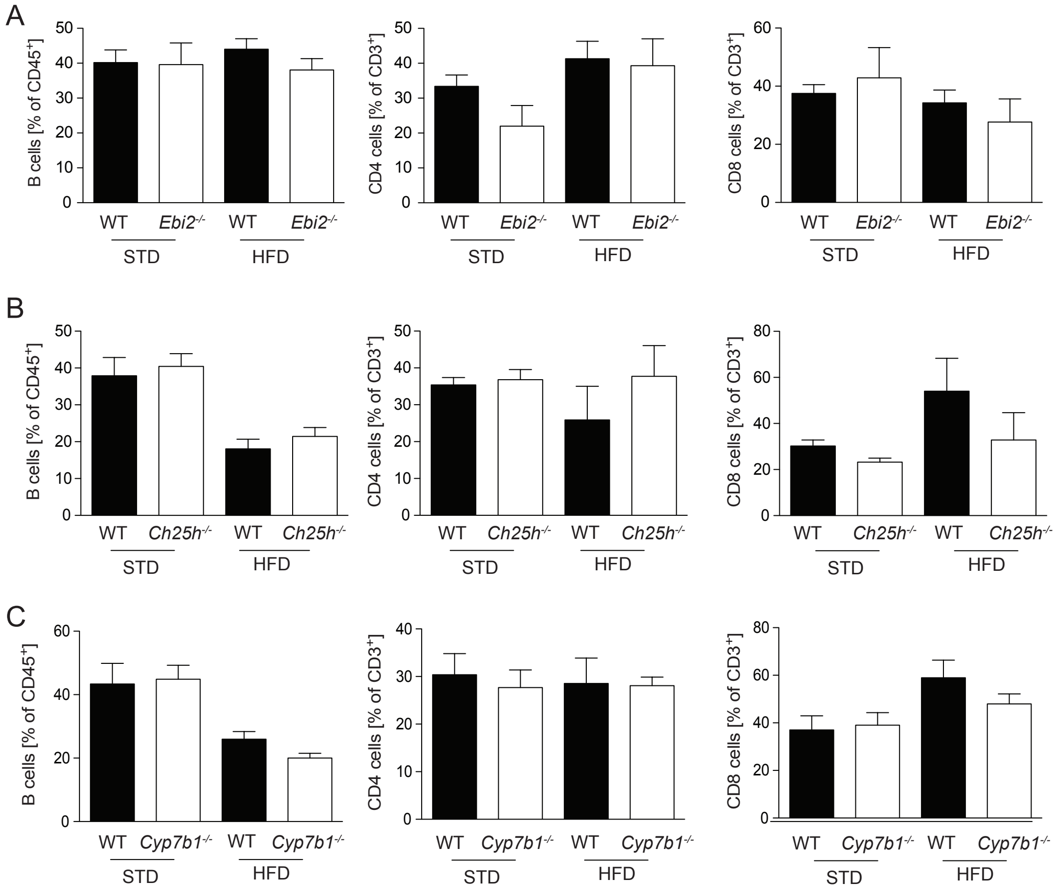

Supplemental Figure S5: No difference in major populations of liver infiltrating cells upon knockout of players of the EBI2 – oxysterol axis in health and HFD feeding. Intrahepatic mononuclear cells were isolated from livers after 20 week HFD or STD and analysed by flow cytometry for B220, CD3, CD4 and CD8 expression. **(A)** *Ebi2*$^{-/-}$, **(B)** *Ch25h*$^{-/-}$ and **(C)** *Cyp7b1*$^{-/-}$ mice and the respective littermate wildtype controls. Mann-Whitney U test (no significant differences detected).

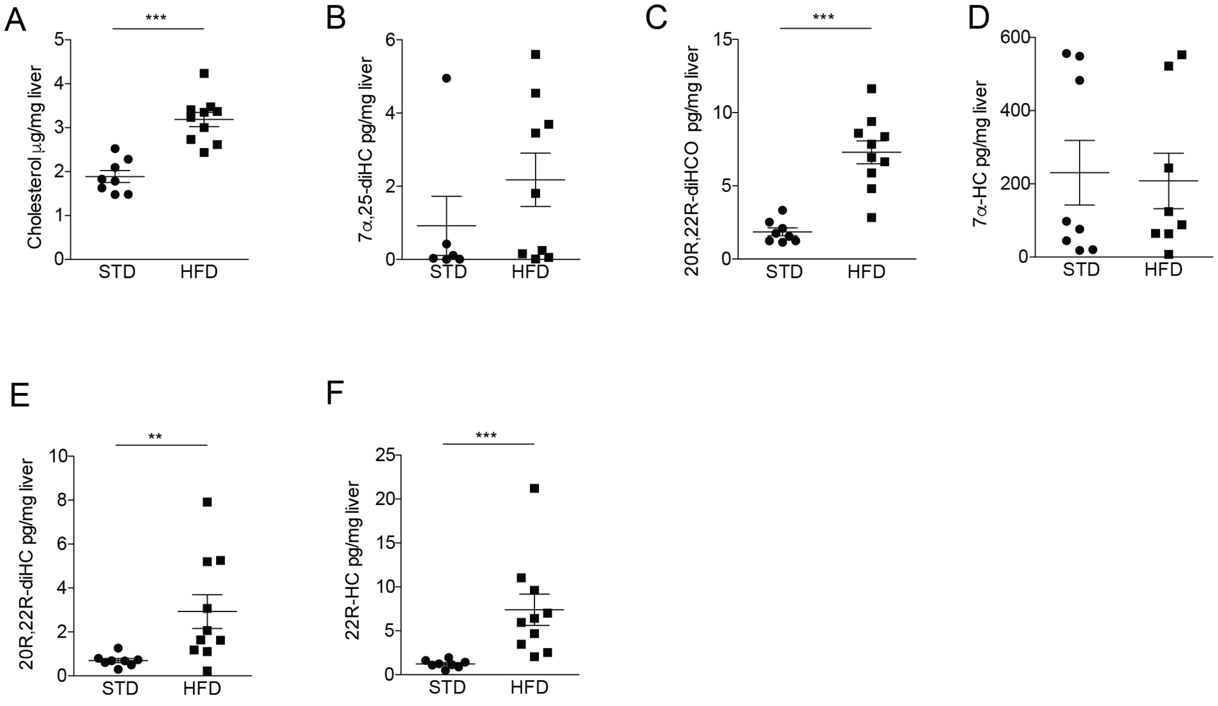

Supplemental Figure S6: Levels of additional oxysterols in liver in a murine model of NAFLD/NASH. 8 week-old male C57BL/6 mice were fed a high fat diet with high fructose corn syrup equivalent (HFD) or standard diet (STD) for 20 weeks. Levels of the indicated oxysterol in liver tissue of HFD and STD controls were measured by LC-MS. Mann-Whitney U test; ***p<0.001, **p<0.01, *p<0.05. (n_{STD} = 8, ≥6 valid data points; n_{HFD} = 10, ≥8 valid data points).

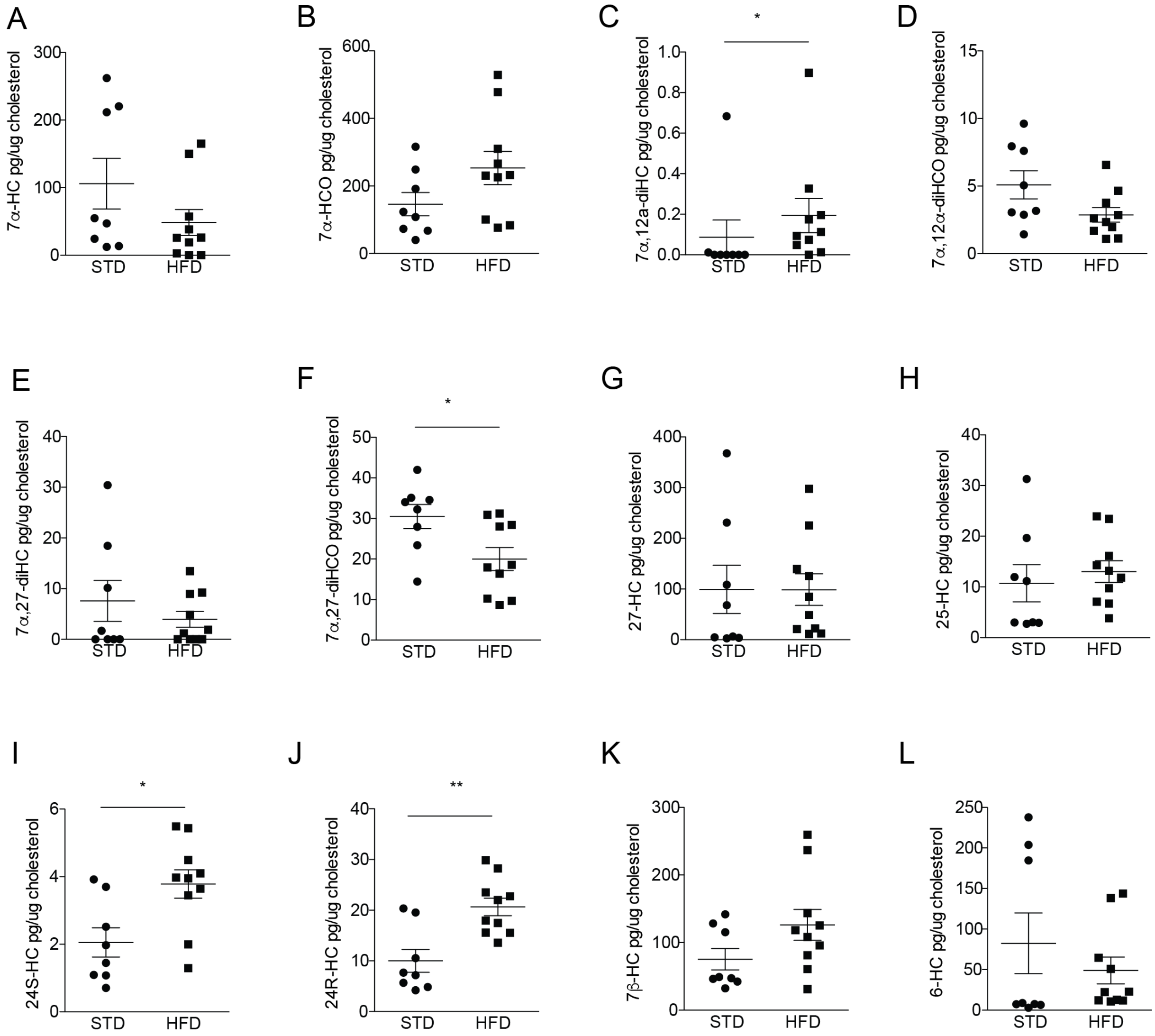

Supplemental Figure S7: Oxysterols levels in a murine model of NAFLD/NASH normalized to cholesterol levels. Oxysterols levels are indicated as in Figure 6. Oxysterol levels were normalized to the cholesterol concentration within the respective samples (pg/µg cholesterol). Mann-Whitney U test. ***p<0.001, **p<0.01, *p<0.05
